# Tau biomarkers in Special Operations Forces with repeated blast exposure: a cross-sectional study

**DOI:** 10.1093/braincomms/fcag061

**Published:** 2026-02-26

**Authors:** Chieh-En Jane Tseng, Jennifer Guo, Natalie Gilmore, Douglas N Greve, Isabella R McKinney, Brian C Healy, Samantha L Tromly, Kristen Dams-O’Connor, Christine L Mac Donald, Daniel P Perl, Jacob M Hooker, Yelena G Bodien, Brian L Edlow, William T Kimberly, Nicole R Zürcher

**Affiliations:** Athinoula A. Martinos Center for Biomedical Imaging, Department of Radiology, Massachusetts General Hospital and Harvard Medical School, Boston, MA 02129, USA; Department of Neurology, Massachusetts General Hospital and Harvard Medical School, Boston, MA 02114, USA; Department of Neurology, Massachusetts General Hospital and Harvard Medical School, Boston, MA 02114, USA; Center for Neurotechnology and Neurorecovery, Massachusetts General Hospital, Boston, MA 02114, USA; Athinoula A. Martinos Center for Biomedical Imaging, Department of Radiology, Massachusetts General Hospital and Harvard Medical School, Boston, MA 02129, USA; Department of Neurology, Massachusetts General Hospital and Harvard Medical School, Boston, MA 02114, USA; Center for Neurotechnology and Neurorecovery, Massachusetts General Hospital, Boston, MA 02114, USA; Biostatistics Center, Massachusetts General Hospital, Boston, MA 02114, USA; Harvard T.H. Chan School of Public Health, Boston, MA 02115, USA; Institute of Applied Engineering, University of South Florida, Tampa, FL 33612, USA; Department of Rehabilitation and Human Performance, Icahn School of Medicine, NewYork, NY 10029, USA; Brain Injury Research Center, Icahn School of Medicine, NewYork, NY 10029, USA; Department of Neurology, Icahn School of Medicine, NewYork, NY 10029, USA; Department of Neurological Surgery, University of Washington, Seattle, WA 98195, USA; Department of Pathology, F. Edward Hébert School of Medicine, Uniformed Services University, Bethesda, MD 20814, USA; Athinoula A. Martinos Center for Biomedical Imaging, Department of Radiology, Massachusetts General Hospital and Harvard Medical School, Boston, MA 02129, USA; Department of Neurology, Massachusetts General Hospital and Harvard Medical School, Boston, MA 02114, USA; Center for Neurotechnology and Neurorecovery, Massachusetts General Hospital, Boston, MA 02114, USA; Department of Physical Medicine and Rehabilitation, Spaulding Rehabilitation Hospital and Harvard Medical School, Charlestown, MA 02129, USA; Athinoula A. Martinos Center for Biomedical Imaging, Department of Radiology, Massachusetts General Hospital and Harvard Medical School, Boston, MA 02129, USA; Department of Neurology, Massachusetts General Hospital and Harvard Medical School, Boston, MA 02114, USA; Center for Neurotechnology and Neurorecovery, Massachusetts General Hospital, Boston, MA 02114, USA; Department of Neurology, Massachusetts General Hospital and Harvard Medical School, Boston, MA 02114, USA; Athinoula A. Martinos Center for Biomedical Imaging, Department of Radiology, Massachusetts General Hospital and Harvard Medical School, Boston, MA 02129, USA

**Keywords:** tau, blood biomarker, neuroimaging

## Abstract

US Special Operations Forces (SOF) personnel endure repeated blasts throughout training and combat. Recent human postmortem, neuroimaging and blood proteomic work suggest that tau pathology is present following repeated blast exposure. This study aimed to determine whether blood tau markers are associated with brain tau paired helical filaments (PHFs) in SOF personnel. Twenty-eight active-duty SOF completed a positron emission tomography–magnetic resonance imaging scan with the PHF-specific radiotracer fluorine-18 MK6240 ([^18^F]MK6240) and provided blood samples to measure total tau and phosphorylated tau181 (p-tau181). Whole brain voxel-wise analysis showed that higher total tau in the blood was associated with higher [^18^F]MK6240 uptake in the left temporal cortex, parahippocampal gyrus and hippocampus. We performed *post hoc* analyses to assess whether brain or blood tau measures were associated with memory performance. Higher levels of blood total tau and p-tau181 were associated with a longer response time and lower throughput (i.e. fewer accurate responses per minute) during the Code Substitution-Delayed test, a visual memory task in the Automated Neuropsychological Assessment Metrics (ANAM). This study provides preliminary evidence in active-duty SOF that blood total tau is associated with regional [^18^F]MK6240 uptake in the brain and that blood total tau and p-tau181 are associated with memory performance.

## Teaser

Military personnel are exposed to repeated blasts that may impact brain health. Tseng *et al*. report that blood total tau is associated with regional brain tau and that blood tau markers are associated with memory performance in active-duty Special Operations Forces personnel, suggesting the involvement of tau in this cohort.

## Graphical Abstract

**Graphical Abstract:**
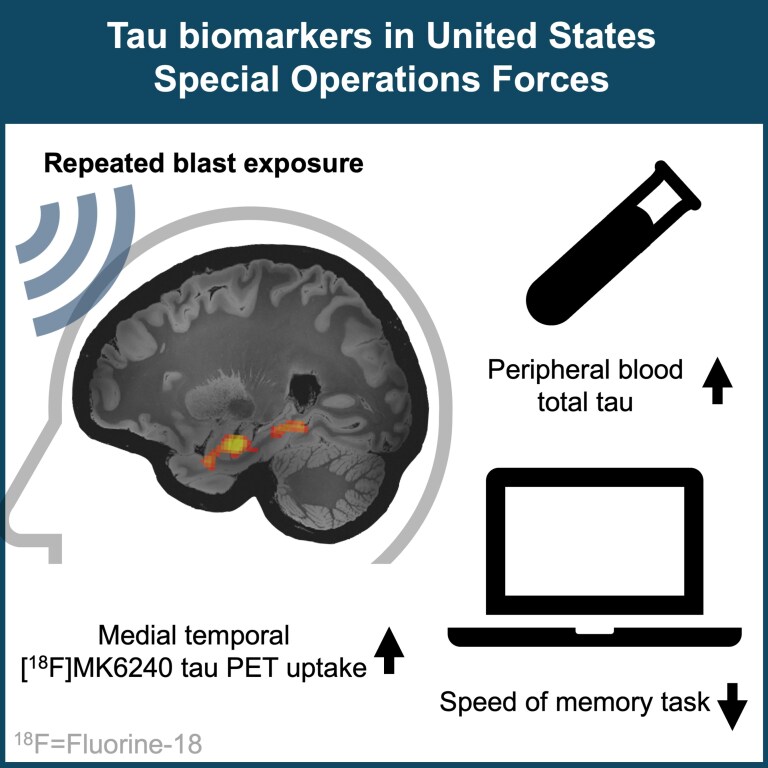

## Introduction

Human postmortem and *in vivo* neuroimaging studies have reported tau-related alterations in military veterans with blast exposure.^1-4^ Postmortem work in military veterans, many of whom also participated in contact sports, has shown abnormal phosphorylated tau (p-tau) aggregates of neurofibrillary tangles (NFTs) in perivascular regions and sulcal depths across the whole brain.^3,4^ In addition, *in vivo* positron emission tomography (PET) with [^18^F]flortaucipir showed that tau signal in frontal and occipital areas and in the cerebellum was positively associated with blast exposure, but not with blunt-impact concussion or symptom duration in military veterans.^2^ However, recent postmortem work found infrequent presence of p-tau in the brains of deceased service members with a history of blast exposure,^5^ suggesting that further investigation is needed to understand the occurrence of tau pathology in relation to military blast exposure.

Tau and p-tau can be assessed peripherally in the blood, an approach that has potential to detect early markers of neurological diseases during life.^6^ With regard to blast exposure, blood tau markers, such as total tau and phosphorylated tau181 (p-tau181), have been shown to increase 72 h following blast exposure larger than 5 pounds per square inch (psi)^7^ and to be elevated in breacher trainees exposed to blasts.^8^ Tau concentrations in neuronal-derived extracellular vesicles are also elevated in experienced breachers.^9^ In the context of Alzheimer’s disease, blood-based tau markers may be associated with the presence of *in vivo* brain tau pathology,^10^ which can be measured with PET and the tau paired helical filament (PHF)-specific radiotracer [^18^F]MK6240.^11^

In a recent pilot study of 30 active-duty US Special Operations Forces (SOF) personnel (ReBlast), we did not observe associations between cumulative blast exposure and blood tau markers or regional [^18^F]MK6240 PET signal.^12^ However, we found that the median levels of total tau and p-tau181 were 10 and 1.5 times higher, respectively, in these active-duty SOF participants (mean age 37 and mean duration of service 17 years) than the levels reported in a separate study of breacher trainees using the same commercial platform.^8,12^ This observation provided the rationale for investigating blood tau and brain PHF in active-duty SOF in the current study. Further, to date, the association between tau blood biomarkers and brain PHF has not been investigated in individuals with high levels of repeated blast exposure. Peripheral blood measures can easily be collected and could potentially aid in initial screening and clinical assessment for blast-associated brain injury. We tested the hypothesis that the blood tau markers total tau and p-tau181 are associated with brain PHF measured by [^18^F]MK6240 PET–magnetic resonance imaging (MRI) in active-duty SOF.

## Materials and methods

### Participants

All participants were enrolled in the ReBlast Pilot study, as previously described (ClinicalTrials.gov NCT05183087).^12,13^ Briefly, ReBlast Pilot included male active-duty SOF personnel with prior combat exposure during deployment, and exposure to explosive blast overpressure, which was assessed using the generalized blast exposure value (GBEV).^14^ Participants were excluded if they had moderate–severe traumatic brain injury or any PET–MRI contraindications. Lifetime history of head impact was assessed using the Brain Injury Screening Questionnaire (BISQ).^15^ Participants were characterized into BISQ high (i.e. those who had more blows to the head than they can remember) and BISQ low (i.e. those who had a finite number of blows to the head) groups. Participants completed a [^18^F]MK6240 PET–MRI scan at the Massachusetts General Hospital Athinoula A. Martinos Center for Biomedical Imaging and provided blood samples for biomarker analyses.

### PET–MRI data processing

PET–MRI processing followed previously published methods.^12^ Briefly, [^18^F]MK6240 PET data from 70–90 min post-radiotracer injection were reconstructed into 5-min standardized uptake value (SUV) frames, motion corrected, averaged and skull stripped. The individual SUV image was registered to the Montreal Neurological Institute (MNI) standard space using the transformation matrix generated from registering the T1-weighted structural scan to the MNI template. The SUV in MNI space was then normalized by the isthmus cingulate cortex (SUV ratio, SUVR), as done previously,^2^ and smoothed using a Gaussian kernel with a full width half maximum of 8 mm.

### Blood tau measurements

Plasma samples were collected from fasted participants and stored at −80°C. Once all samples were collected, total tau and p-tau181 were measured using the Simoa Human Neurology 4-Plex A (N4PA) and pTau181 assays at the Simoa Accelerator Laboratory (Quanterix, Billerica, MA).

### Memory-related cognitive assessments

Selected memory-related cognitive measures collected in ReBlast Pilot as part of a large battery of neurobehavioral assessments^13^ were used in the *post hoc* analysis of this study. These include the delayed recall trial of the Rey Auditory Verbal Learning Test^16^ (RAVLT, verbal memory) and Rey–Osterrieth Complex Figure Test^17,18^ (ROCFT, visual memory), and the mean response time and throughput (accurate responses per minute) of the Code Substitution Delayed (CDD) and Memory Search tasks in the Automated Neuropsychological Assessment Metrics^19^ (ANAM), a cognitive assessment tool routinely used by the US military. The CDD measures delayed visual memory and learning, while Memory Search assesses working memory. We used raw scores in our analyses.

### Statistical analysis

We performed whole brain voxel-wise analyses using a general linear model with age as a covariate to assess the association between [^18^F]MK6240 SUVR and either total tau or p-tau181 across the whole brain [FMRIB’s software library (FSL) tool FEAT]. The statistical significance threshold was set at Z > 2.3, *P*_cluster_ < 0.05. Statistical tests were performed in MATLAB version R2018b. Multivariable linear regression models were used to assess the association between tau markers (brain and blood tau) and memory-related cognitive measures, controlling for age. Variables were log transformed when needed to ensure that the residuals of each multivariable linear regression model were normally distributed. Mann–Whitney U-tests were used to assess differences in blood tau and [^18^F]MK6240 SUVR uptake measures between BISQ high and low groups.

## Results

### Study participants

A flow diagram of the screening and enrolment of participants was previously reported.^12^ Of the 30 participants meeting inclusion criteria, 28 completed a [^18^F]MK6240 PET–MRI scan (Supplementary Fig. 1). Two participants did not complete a [^18^F]MK6240 PET–MRI scan due to radiotracer production failure. All participants provided blood samples for blood biomarker analysis, including total tau and p-tau181. The participants who both completed a [^18^F]MK6240 PET–MRI scan and provided blood samples were included here (i.e. *N* = 28). See Table 1 for demographics of the 28 participants with both blood tau and [^18^F]MK6240 PET-MRI data, information on blast exposure, blood tau and radiochemistry measures.

**Table 1:** Demographic, blast exposure, blood tau, radiochemistry and cognitive measures

N	28
Demographics	
Age (years)	37.0 ± 4.1 (29–43)
Sex	28 M
Weight (kg)	88.9 ± 10.2 (63.5–111.1)
Years of education	16.7 ± 2.0 (14–21)
Years in service	17.1 ± 4.5 (8–26)
Blast exposure	
GBEV	9 902 540 (387 861–363 812 869)
Blast recency	Within 1 year, *N* = 24 (85.7%) >1 year, *N* = 2 (7.1%) >2 years, *N* = 2 (7.1%)
Head impact	
Number of blows to the head	BISQ high: 20 BISQ low: 8
Blood tau biomarkers	
Total tau (pg/mL)	4.9 ± 4.0 (0.3–17.7)
p-tau181 (pg/mL)	2.1 ± 1.7 (1.0–10.1)
[^18^F]MK6240 PET radiochemistry	
Injected dose (mCi)	5.4 ± 0.3 (4.9–5.9)
Molar activity (mCi/nmol)	4.2 ± 4.0 (0.6–12.9)
Memory-related cognitive measures	
RAVLT, delayed recall	10.8 ± 3.8 (0.0–15.0)
ROCFT, delayed recall	19.8 ± 6.1 (8.5–34.0)
ANAM CDD, response time (ms)	1313.9 ± 545.8 (763.0–2694.9)
ANAM CDD, throughput (accurate response/min)	47.3 ± 17.7 (16.1–78.6)
ANAM Memory Search, response time (ms)	789.2 ± 157.0 (536.1–1135.4)
ANAM Memory Search, throughput (accurate response/min)	77.3 ± 17.3 (45.7–111.9)

Continuous variables are presented as mean ± SD (range). The generalized blast exposure value (GBEV) is presented as median (range). Participants in the BISQ high group reported more blows to the head than they can remember. Participants in the BISQ low group reported a finite number of blows to the head (range: 1–13). The range of scores possible for the RAVLT is 0–15, and for ROCFT, it is 0–36.

ANAM = Automated Neuropsychological Assessment Metrics, BISQ = Brain Injury Screening Questionnaire, CDD = Code Substitution Delayed, M = male, RAVLT = Rey Auditory Verbal Learning Test, ROCFT = Rey–Osterrieth Complex Figure Test.

### Association between total tau and [^18^F]MK6240 uptake

**Figure 1:**
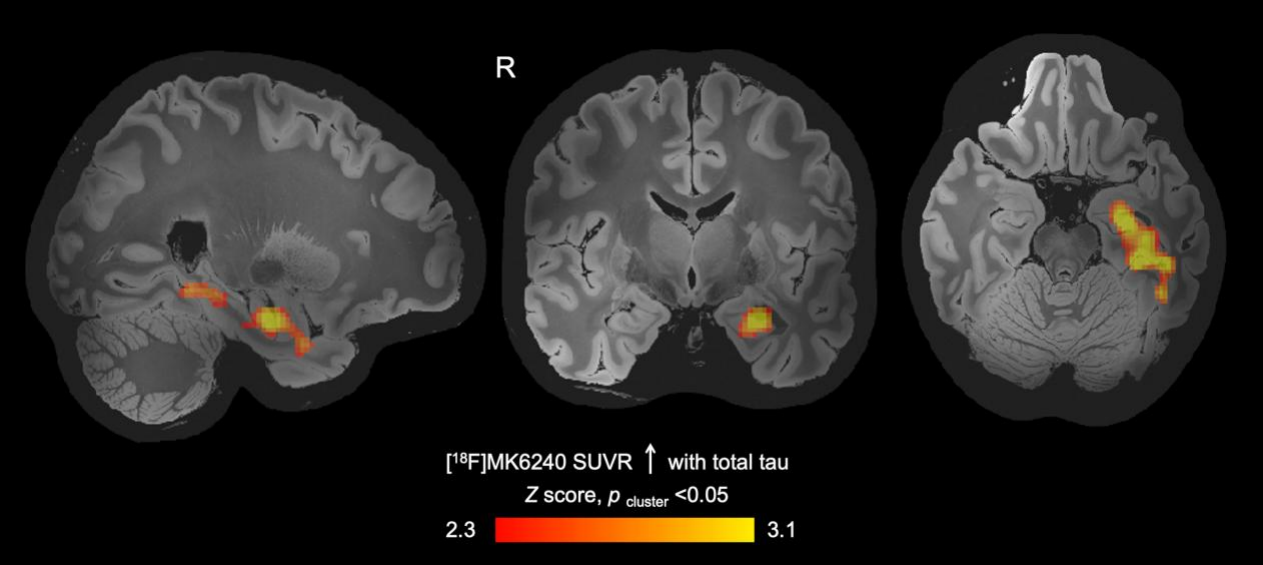
**Relationship between brain PHF tau measured by [^18^F]MK6240 PET and blood total tau in active-duty SOF**. Blood total tau levels were positively associated with PHF as measured by [^18^F]MK6240 SUVR in a region encompassing the left temporal cortex, left parahippocampal gyrus and left hippocampus. Image shows a statistical map of linear regression with total tau and [^18^F]MK6240 SUVR, controlling for age [threshold of *Z* > 2.3, beta = 1.23 × 10^−2^, 95% CI (1.22 × 10^−2^, 1.23 × 10^−2^), *P_cluster_* < 0.05], overlaid on a 500-micron quantitative multi-echo flash template in MNI space.^20^ R = right, SUVR = standardized uptake value ratio, PHF = paired helical filament.

Higher total tau was associated with a higher [^18^F]MK6240 SUVR in a cluster of voxels that included the left temporal cortex, parahippocampal gyrus and hippocampus [beta = 1.23 × 10^−2^, 95% CI (1.22 × 10^−2^, 1.23 × 10^−2^), *P*_cluster_ < 0.05, Fig. 1]. [^18^F]MK6240 SUVR in this *post hoc* region was 0.96 ± 0.07. The volume of the *post hoc* region was not associated with [^18^F]MK6240 SUVR in the *post hoc* region or total tau (Supplementary Results 1.1). Total tau was also positively associated with [^18^F]MK6240 SUVR in anatomically defined hippocampus, parahippocampal gyrus and entorhinal cortex (Supplementary Fig. 2). No brain region showed a negative association between total tau and [^18^F]MK6240 SUVR.

### Association between p-tau181 and [^18^F]MK6240 uptake

No brain region showed a positive association between p-tau181 and [^18^F]MK6240 SUVR. Higher p-tau181 was associated with a lower [^18^F]MK6240 SUVR in a cluster of voxels, including the bilateral cerebellum (Supplementary Fig. 3). [^18^F]MK6240 SUVR in this *post hoc* region was 1.09 ± 0.15. The volume of the *post hoc* region was not associated with [^18^F]MK6240 SUVR in the *post hoc* region and was negatively associated with p-tau181 (Supplementary Results 1.2).

### *Post hoc* analysis—associations of total tau and p-tau181 with memory-related cognitive measures

Given that blood total tau was associated with [^18^F]MK6240 SUVR in brain regions that underlie memory, such as the left temporal cortex, parahippocampal gyrus and hippocampus,^21^ we assessed relationships with psychometric tests of memory that were collected in ReBlast Pilot and report the descriptive statistics in Table 1.

Total tau was positively associated with the response time [beta = 72.87 SUVR/ms, 95% CI (29.27, 116.46), *P* = 0.002] and negatively associated with throughput (accurate responses per minute) [beta = −1.67 SUVR/accurate responses per minute, 95% CI (−3.12, −0.21), *P* = 0.03] for the CDD test, and no other memory measure. P-tau181 was positively associated with the response time [beta = 1181.06 pg/mL/ms, 95% CI (369.62, 1992.49), *P* = 0.006] and negatively associated with throughput (accurate responses per minute) [beta = −3.66 pg/mL/accurate responses per minute, 95% CI (−7.05, −0.26), *P* = 0.04] for the CDD test, and no other memory measure.

### *Post hoc* analysis—association of [^18^F]MK6240 SUVR with memory-related cognitive measures

Our data suggested a possible association between higher [^18^F]MK6240 SUVR in the *post hoc* region and a longer CDD response time [beta = 2832.40 SUVR/ms, 95% CI (−0.81, 5665.61), *P* = 0.05]. [^18^F]MK6240 SUVR in the *post hoc* region was not associated with any other memory measure.

### Assessment of the potential impact of blows to the head

The blood tau measures (total tau and p-tau181) were not different between the BISQ high and low groups (total tau: U = 52, *P* = 0.16; p-tau181: U = 79.5, *P* = 1). [^18^F]MK6240 SUVR in the *post hoc* regions where blood total tau or p-tau181 were associated with [^18^F]MK6240 uptake were not different between the BISQ high and low groups (total tau: U = 75, *P* = 0.82; p-tau181: U = 68, *P* = 0.56).

## Discussion

In this prospective observational study of the relationship between blood tau biomarkers and brain PHF in active-duty US SOF personnel, we found a positive association between total tau and [^18^F]MK6240 SUVR in an area including the left temporal cortex, left parahippocampal gyrus and left hippocampus, brain regions relevant for memory.^21^  *Post hoc* analyses revealed that higher blood total tau and p-tau181 were associated with a longer response time and lower throughput (i.e. fewer accurate responses per minute) on a delayed visual memory task. Collectively, these observations indicate an association between higher blood tau levels and higher brain PHF in regions relevant for memory and that elevations in blood tau levels may be associated with slower visual memory response times in active-duty SOF personnel.

Higher blood total tau was associated with higher brain PHF as measured by [^18^F]MK6240 SUVR in the left temporal gyrus, parahippocampal gyrus and hippocampus. This finding suggests that future work is warranted to investigate whether blood tau measures, which are more easily obtained than are brain PHF measures, can be used as a screening tool to identify individuals who may have tau-associated neurobiological changes. Moreover, the medial temporal lobe emerged as a region that may have tau-related changes following repeated blast exposure, which we did not find in our previous work given that medial temporal lobe regions were not included in the *a priori* regions for [^18^F]MK6240 PET.^12^ Surprisingly, we observed a negative association between p-tau181 and [^18^F]MK6240 SUVR in the cerebellum, with higher p-tau181 being associated with lower [^18^F]MK6240 SUVR. The potential relevance of this association is uncertain. Larger cross-sectional and longitudinal studies will be required to understand the implications of these neurobiological alterations and the timing of these events.

Higher levels of blood total tau and p-tau181 were associated with a longer response time and less accurate responses per minute for the same delayed visual memory task. This visual memory task on the ANAM was the only measure that was associated with blood tau measures, suggesting its potential utility to detect cognitive changes that are related to tau in active-duty SOF.

Given that our study did not include a control group, we are unable to determine whether blood tau markers or [^18^F]MK6240 SUVR are altered in SOF personnel compared to a non-SOF control group. Compared to prior studies of civilians, athletes and breacher trainees that used similar assays,^8,22-24^ the blood tau values were consistently higher in our SOF cohort. However, these control groups differ not only in terms of repeated blast exposure but also in exposure to combat stress, as well as cognitive and physical factors.^12^ Future longitudinal studies of SOF personnel may be the most reliable approach to assess changes in brain health in relation to ongoing blast exposure.

In terms of brain PHF tau measured by [^18^F]MK6240 PET, no significant cerebral regional elevations were observed based on visual inspection of SUVR images (see Supplementary Fig. 3). In contrast, in Alzheimer’s disease, increased [^18^F]MK6240 uptake has been reported at the individual level.^25,26^ [^18^F]MK6240 is known to bind to mixed 3-repeat/4-repeat tau, which is found in Alzheimer’s disease, but may not be the main tau isoform in other tauopathies.^27^ Notably, the tau distribution observed by our group and others in military cohorts with blast exposure through PET imaging^2^ is not consistent with the tau distribution in Alzheimer’s disease^28^ and chronic traumatic encephalopathy.^29^ Given the exploratory nature of this pilot study, we did not correct for multiple comparisons, and the results from this work will need to be replicated in larger-scale studies. Future work will benefit from longitudinal investigations to assess temporal alterations in tau pathology in SOF personnel.

Our study suggests that total tau is a potential blood biomarker for the presence of brain PHF in active-duty SOF. We also provide preliminary evidence that higher blood total tau and p-tau181 are linked with slower memory performance. Future studies that monitor longitudinal tau measures, in blood and the brain, and that test for memory changes, are required to understand the underlying pathophysiology and associated cognitive outcomes in SOF personnel.

## Supplementary Material

fcag061_Supplementary_Data

## Data Availability

USSOCOM policy limits public sharing of data generated and analysed for the current study. Any future requests for these data may be submitted to the corresponding author for subsequent vetting and approval by USSOCOM.

## References

[1] Dickstein DL, De Gasperi R, Gama Sosa MA, et al Brain and blood biomarkers of tauopathy and neuronal injury in humans and rats with neurobehavioral syndromes following blast exposure. Mol Psychiatry. 2021;26(10):5940–5954. 32094584 10.1038/s41380-020-0674-z PMC7484380

[2] Robinson ME, McKee AC, Salat DH, et al Positron emission tomography of tau in Iraq and Afghanistan veterans with blast neurotrauma. Neuroimage Clin. 2019;21:101651. 30642757 10.1016/j.nicl.2019.101651 PMC6412062

[3] Goldstein LE, Fisher AM, Tagge CA, et al Chronic traumatic encephalopathy in blast-exposed military veterans and a blast neurotrauma mouse model. Sci Transl Med. 2012;4(134):134ra60. 10.1126/scitranslmed.3003716 PMC3739428 22593173

[4] Omalu B, Hammers JL, Bailes J, et al Chronic traumatic encephalopathy in an Iraqi war veteran with posttraumatic stress disorder who committed suicide. Neurosurg Focus. 2011;31(5):E3. 10.3171/2011.9.FOCUS11178 22044102

[5] Priemer DS, Iacono D, Rhodes CH, Olsen CH, Perl DP. Chronic traumatic encephalopathy in the brains of military personnel. N Engl J Med. 2022;386(23):2169–2177. 35675177 10.1056/NEJMoa2203199

[6] Karikari TK, Ashton NJ, Brinkmalm G, et al Blood phospho-tau in Alzheimer disease: Analysis, interpretation, and clinical utility. Nat Rev Neurol. 2022;18(7):400–418. 35585226 10.1038/s41582-022-00665-2

[7] Edwards KA, Leete JJ, Tschiffely AE, et al Blast exposure results in tau and neurofilament light chain changes in peripheral blood. Brain Inj. 2020;34(9):1213–1221. 32755419 10.1080/02699052.2020.1797171

[8] Vorn R, Naunheim R, Lai C, Wagner C, Gill JM. Elevated axonal protein markers following repetitive blast exposure in military personnel. Front Neurosci. 2022;16:853616. 35573288 10.3389/fnins.2022.853616 PMC9099432

[9] Edwards KA, Greer K, Leete J, et al Neuronally-derived tau is increased in experienced breachers and is associated with neurobehavioral symptoms. Sci Rep. 2021;11(1):19527. 34593828 10.1038/s41598-021-97913-0 PMC8484560

[10] Tissot C, Therriault J, Kunach P, et al Comparing tau status determined via plasma pTau181, pTau231 and [(18)F]MK6240 tau-PET. EBioMedicine. 2022;76:103837. 35134647 10.1016/j.ebiom.2022.103837 PMC8844756

[11] Aguero C, Dhaynaut M, Normandin MD, et al Autoradiography validation of novel tau PET tracer [F-18]-MK-6240 on human postmortem brain tissue. Acta Neuropathol Commun. 2019;7(1):37. 30857558 10.1186/s40478-019-0686-6 PMC6410510

[12] Gilmore N, Tseng C-EJ, Maffei C, et al Impact of repeated blast exposure on active-duty United States special operations forces. Proc Natl Acad Sci U S A. 2024;121(19):e2313568121. 38648470 10.1073/pnas.2313568121 PMC11087753

[13] Edlow BL, Bodien YG, Baxter T, et al Long-term effects of repeated blast exposure in United States special operations forces personnel: A pilot study protocol. J Neurotrauma. 2022;39(19–20):1391–1407. 35620901 10.1089/neu.2022.0030 PMC9529318

[14] Modica LCM, Egnoto MJ, Statz JK, Carr W, Ahlers ST. Development of a blast exposure estimator from a department of defense-wide survey study on military service members. J Neurotrauma. 2021;38(12):1654–1661. 33138683 10.1089/neu.2020.7405

[15] Dams-O'Connor K, Cantor JB, Brown M, Dijkers MP, Spielman LA, Gordon WA. Screening for traumatic brain injury: Findings and public health implications. J Head Trauma Rehabil. 2014;29(6):479–489. 25370440 10.1097/HTR.0000000000000099 PMC4985006

[16] Rey A . L'examen clinique en psychologie [the clinical psychological examination]. *Presses Universitaires de France*; 1964.

[17] Osterrieth PA . Le test de copie d'une figure complexe; contribution a l'etude de la perception et de la memoire. Arch Psychol (Geneve). 1944;30:206–356.

[18] Rey A . L'examen psychologique dans les cas d'encephalopathie traumatique. Arch Psychol (Geneve). 1941;28:286–340.

[19] Vincent AS, Roebuck-Spencer T, Gilliland K, Schlegel R. Automated neuropsychological assessment metrics (v4) traumatic brain injury battery: Military normative data. Mil Med. 2012;177(3):256–269. 22479912 10.7205/milmed-d-11-00289

[20] Edlow BL, Mareyam A, Horn A, et al 7 tesla MRI of the ex vivo human brain at 100 micron resolution. Sci Data. 2019;6(1):244. 31666530 10.1038/s41597-019-0254-8 PMC6821740

[21] Squire LR, Stark CEL, Clark RE. The medial temporal lobe. Annu Rev Neurosci. 2004;27:279–306. 15217334 10.1146/annurev.neuro.27.070203.144130

[22] Cooper JG, Stukas S, Ghodsi M, et al Age specific reference intervals for plasma biomarkers of neurodegeneration and neurotrauma in a Canadian population. Clin Biochem. 2023;121–122:110680. 10.1016/j.clinbiochem.2023.110680 37884086

[23] Asken BM, Yang Z, Xu H, et al Acute effects of sport-related concussion on serum glial fibrillary acidic protein, ubiquitin C-terminal hydrolase L1, total tau, and neurofilament light measured by a multiplex assay. J Neurotrauma. 2020;37(13):1537–1545. 32024456 10.1089/neu.2019.6831

[24] Lanz TA, Ruprecht K, Somps CJ, et al Longitudinal evaluation of serum neurofilament light levels in normal healthy volunteers: Defining a threshold of concern. J Neurol. 2025;272(8):512. 40665000 10.1007/s00415-025-13246-2 PMC12263756

[25] Lois C, Gonzalez I, Johnson KA, Price JC. PET imaging of tau protein targets: A methodology perspective. Brain Imaging Behav. 2019;13(2):333–344. 29497982 10.1007/s11682-018-9847-7 PMC6119534

[26] Pascoal TA, Shin M, Kang MS, et al In vivo quantification of neurofibrillary tangles with [(18)F]MK-6240. Alzheimers Res Ther. 2018;10(1):74. 30064520 10.1186/s13195-018-0402-y PMC6069775

[27] Malarte M-L, Gillberg P-G, Kumar A, Bogdanovic N, Lemoine L, Nordberg A. Discriminative binding of tau PET tracers PI2620, MK6240 and RO948 in Alzheimer’s disease, corticobasal degeneration and progressive supranuclear palsy brains. Mol Psychiatry. 2023;28(3):1272–1283. 36447011 10.1038/s41380-022-01875-2 PMC10005967

[28] Cho H, Choi JY, Hwang MS, et al In vivo cortical spreading pattern of tau and amyloid in the Alzheimer disease spectrum. Ann Neurol. 2016;80(2):247–258. 27323247 10.1002/ana.24711

[29] McKee AC, Stein TD, Kiernan PT, Alvarez VE. The neuropathology of chronic traumatic encephalopathy. Brain Pathol. 2015;25(3):350–364. 25904048 10.1111/bpa.12248 PMC4526170

