# Supplementary material for: Tau biomarkers in Special Operations Forces with repeated blast exposure: a cross-sectional study
Source: Brain Commun. 2026 Feb 26;8(2):fcag061. doi: 10.1093/braincomms/fcag061 (PMC12967882; doi:10.1093/braincomms/fcag061)
Supplement: fcag061_Supplementary_Data [file fcag061_supplementary_data.docx]

**Supplementary Material**

**Disclaimer:** The views expressed in this manuscript are entirely those of the authors and do not necessarily reflect the views, policy, or position of the United States Government, Department of Defense, United States Special Operations Command, or the Uniformed Services University of the Health Sciences.

**Supplementary Results**

- 1. **Volumetric analysis of the posthoc region where total tau was associated with [^18^F]MK6240 uptake**

The volume of the posthoc region where blood total tau was associated with [^18^F]MK6240 SUVR was extracted. The volume of the posthoc region was not associated with [^18^F]MK6240 SUVR in the posthoc region (p=0.84) or total tau (p=0.54), controlling for age and estimated total intracranial volume (eTIV).

- 1. **Volumetric analysis of the posthoc region where p-tau181 was associated with [^18^F]MK6240 uptake**

The volume of the cerebellar posthoc region where p-tau181 was associated with [^18^F]MK6240 SUVR was extracted. The volume of the posthoc region was not associated with [^18^F]MK6240 SUVR in the posthoc region, controlling for age and eTIV (p>0.05). The volume of the posthoc region was negatively associated with p-tau181 (p=0.02), controlling for age and eTIV. This finding indicates that Special Operations Forces personnel with a smaller volume of the cerebellar posthoc region had higher levels of p-tau181.

**Supplementary Figures**


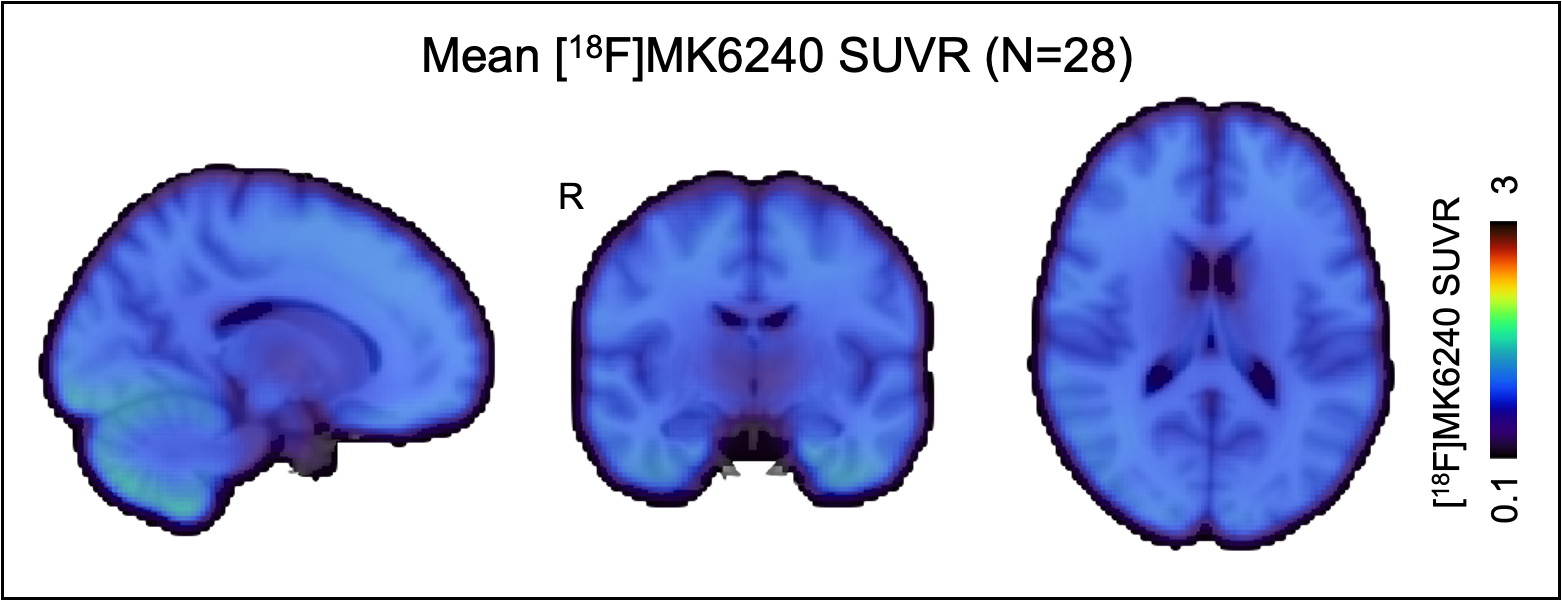


**Supplementary Figure 1.** Group average [^18^F]MK6240 SUVR map, 70-90 minutes post-radiotracer injection, overlaid on the MNI template. SUVR=standardized uptake value ratio, MNI=Montreal Neurological Institute.


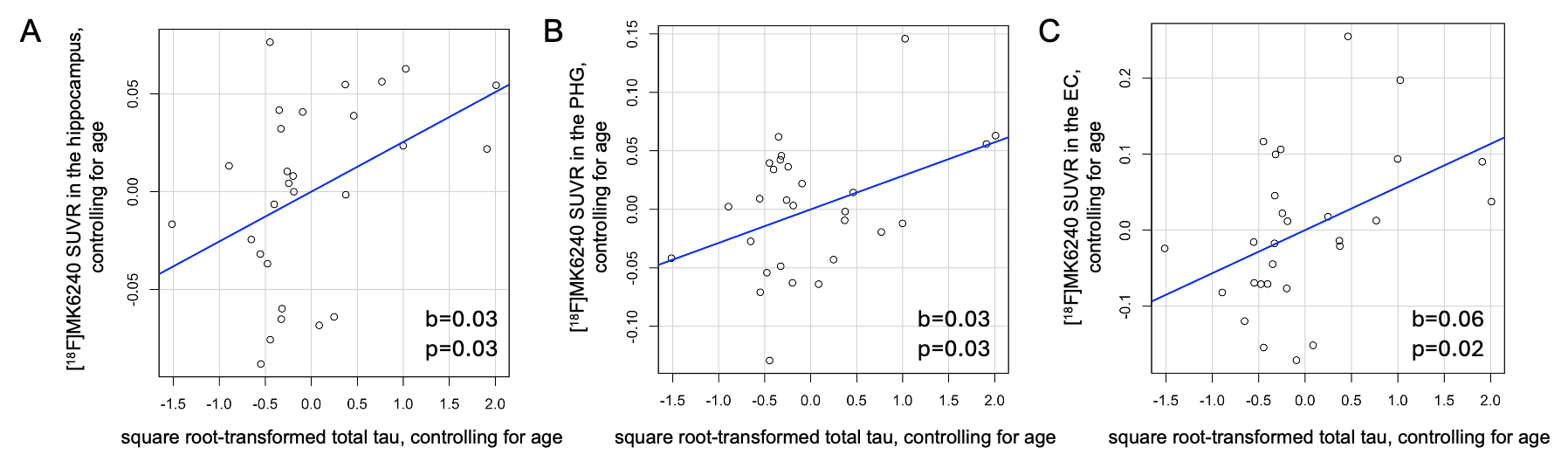


**Supplementary Figure 2.** Partial regression plots between blood total tau and [^18^F]MK6240 uptake in anatomically defined medial temporal lobe regions while controlling for age. Residuals of [^18^F]MK6240 uptake in the hippocampus (A), parahippocampal gyrus (B), and entorhinal cortex (C) after controlling for age are plotted against the residuals of square-root-transformed blood total tau after controlling for age. SUVR=standardized uptake value ratio, EC=entorhinal cortex, PHG=parahippocampal gyrus.


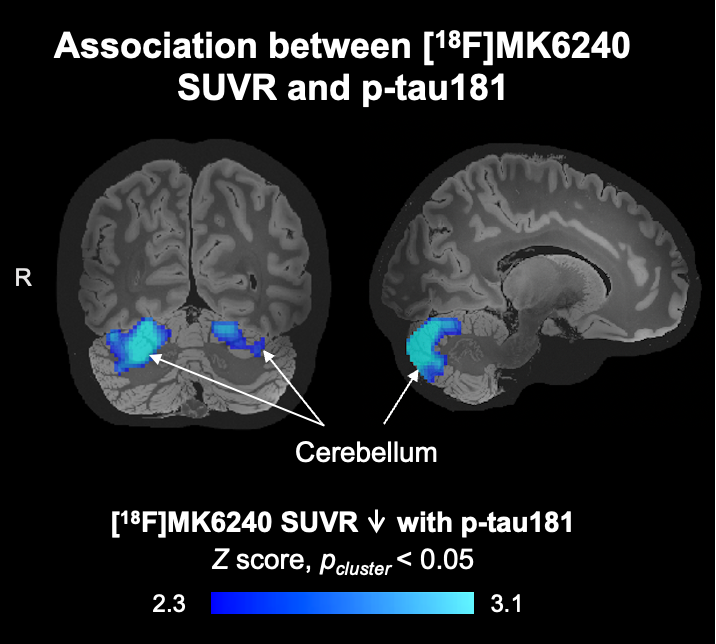


**Supplementary Figure 3.** Blood p-tau181 levels were negatively associated with *in vivo* tau pathology as measured by [^18^F]MK6240 SUVR in the cerebellum. Image shows a statistical map of linear regression with p-tau181 and [^18^F]MK6240 SUVR, controlling for age (threshold of *Z*>2.3, beta=-5.02x10^-2^, 95% CI[-5.08x10^-2^, -4.97x10^-2^], *p_cluster_*<0.05), overlaid on a 500-micron quantitative multi-echo flash template in MNI space. SUVR=standardized uptake value ratio, MNI=Montreal Neurological Institute.

**Data codes**

Multivariable linear regressions were performed using the fitlm MATLAB function and Mann-Whitney U tests were performed using the ranksum MATLAB function. Partial regression plots were generated using the avPlots function in R.
